# Studies of probe tip materials by atomic force microscopy: a review

**DOI:** 10.3762/bjnano.13.104

**Published:** 2022-11-03

**Authors:** Ke Xu, Yuzhe Liu

**Affiliations:** 1 School of Electrical & Control Engineering, Shenyang Jianzhu University, Shenyang 110168, China

**Keywords:** AFM, carbon nanotube probe, colloid probe, metal probe

## Abstract

As a tool that can test insulators' surface morphology and properties, the performance index of atomic force microscope (AFM) probes is the most critical factor in determining the resolution of microscopy, and the performance of probes varies in various modes and application requirements. This paper reviews the latest research results in metal, carbon nanotube, and colloidal probes and reviews their related methods and techniques, analyses the advantages and disadvantages of the improved probes compared with ordinary probes by comparing the differences in spatial resolution, sensitivity, imaging, and other performance aspects, and finally provides an outlook on the future development of AFM probes. This paper promotes the development of AFM probes in the direction of new probes and further promotes the broader and deeper application of scanning probe microscope (SPM).

## Introduction

AFM represents a well-established technique for the investigation of the nanosurface morphology. Applications range from mechanical and electrical sample characterization to measurements in liquids [1–4] and even in living cells [5–8]. The critical element of an AFM is the scanning probe itself, which is usually represented by a micro-cantilever beam equipped with a conical scanning tip. As the tip approaches the sample surface, an interaction force is generated that deflects (bends or stretches) the probe cantilever. As the AFM probe moves across the sample surface (in the X and Y directions), morphological information is obtained over the entire scan area. Its tip structure and the mechanical properties of the cantilever beam directly affect the performance, measurement resolution, and image quality of the AFM instrument.

AFM probe tips [9–10] are generally fabricated with coatings, carbon nanotubes, magnetic nanoparticles, or even protein functionalization. A combination of probe resolution and durability or wear and tear and conductivity requirements must be considered before selecting a probe. Metal-coated probes are generally suitable for high-resolution or high conductivity test experiments. Metal nanoclusters adsorbed on two-dimensional materials grown on metal substrates are an attractive platform, offering more possibilities for sharpening tips in a controlled manner. These metal clusters can be easily generated with various metals (e.g., Ir, Au, Ni, Co, Pb, or Sn), allowing the generation of nano-tips with different functionalities. The good point is that these clusters were shown to be removed one by one from the sample surface by tip indentation of the scanning tunneling microscope (STM). The probing of the interaction forces by AFM and thus the analysis of van der Waals (vdW) forces can provide valuable information on the evolution of the tip size.

Carbon nanotube probes have large aspect ratios and good wear resistance. They thus can better avoid artefacts and more accurately reflect the proper shape of the sample at steep locations, making them suitable for high-resolution test experiments. However, previous methods to fabricate carbon nanotube probes are complicated and poorly controlled. Cheng et al. [11] introduced a method to selectively prepare individual carbon nanotubes on AFM tips by controlling the trigger threshold to regulate the growth solution on the tip. The obtained carbon nanotube probes are of suitable length and do not require a subsequent cutting process. Complex nanostructures with high spreading chord ratios can be scanned using carbon nanotube probes, thus solving the long-standing problem of mapping complex nanostructures.

A colloidal probe [12–13] consists of colloidal particles attached to an AFM cantilever to measure the interaction force between the particle and the surface. A new colloidal AFM probe was proposed by Daboss et al. [14]. These conductive spherical boron-doped diamond (BDD)-AFM probes allow electrochemical force spectroscopy. The physical robustness of these bifunctional probes and the excellent electrochemical properties of BDD make this concept a uniquely versatile tool for a variety of scanning probe studies.

This paper reviews the latest research results in the fields of metal probes, carbon nanotube probes, and colloidal probes, reviews their related methods and techniques and analyses the advantages and disadvantages of the improved probes compared with ordinary probes by comparing the differences in performance in terms of spatial resolution, sensitivity, and imaging, and finally provides an outlook on the future development of AFM probes.

## Review

### Metal probe

#### Metal nanocluster probe

Metal nanoclusters contain a few to several hundred atoms that fill the gaps between nanoparticles and molecular compounds and often exhibit molecule-like electrical and optical properties because their size is close to the Fermi wavelength of electrons [15–17]. Metal nanoclusters have size-dependent luminescence properties, and thus the fluorescence emission can be tuned by controlling different sizes. Due to their unique electronic, physical, and optical properties, metal nanoclusters have attracted great interest in recent years for electronic devices, catalysis, bioimaging, and chemical sensing [18–21]. In particular, metal nanoclusters exhibit excellent photostability, large Stokes shifts, and low toxicity compared to quantum dots and organic dyes. Researchers increasingly use them in analytical detection fields such as metal ions, small biological molecules, drug delivery, and bioimaging [22–24].

**Figure 1:**
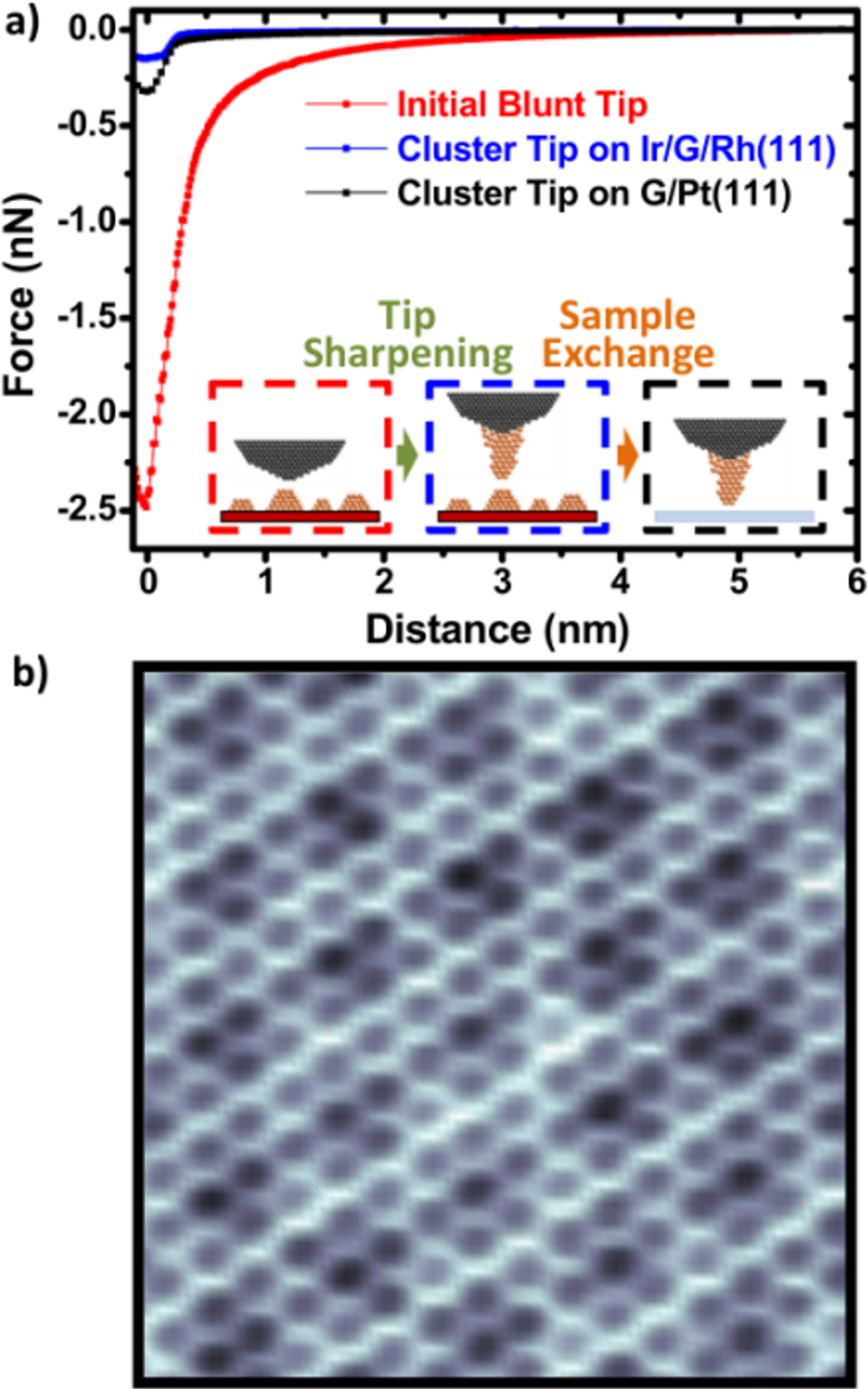
Sharpening of a blunt tip and study of its stability after a macroscopic sample exchange. (a) *F*(z) curves corresponding to the milestones in the sharpening process. The red curve corresponds to the initial blunt tip. The blue curve, measured in the same Ir/G/Rh(111) sample, shows the decrease of the vdW force after the sharpening with clusters. The black curve is obtained with the same tip after exchanging the sample with a G/Pt(111) surface. Acquisition parameters: *f*_o_ = 168274 Hz; *k* = 33.3 N/m; red curve: *V*_CPD_ = −0.1 V; *A* = 13.5 nm. Blue curve: *V*_CPD_ = −0.45 V; *A* = 16 nm. Black curve: *V*_CPD_ = +0.2 V; *A* = 19.2 nm. (b) Atomically resolved NC-AFM image of the G/Pt(111) surface obtained with the cluster tip. Image size: 3.5 × 3.5 nm^2^. Imaging parameters: *A* =14.5 nm; *V*_CPD_ = +0.18 V; Δ*f* = −5.5 Hz. Figure 1 was reproduced from [25] (© 2021 M. D. Jiménez-Sánchez et al., published by Elsevier, distributed under the terms of the Creative Commons Attribution NonCommercial-NoDerivatives 4.0 International License, https://creativecommons.org/licenses/by-nc-nd/4.0/). This content is not subject to CC BY 4.0.

Jiménez-Sánchez et al. [25] studied the extraction of Ir clusters from graphene (by indentation) on the surface of Rh(111) to fabricate AFM sharp nano-tips, i.e., with weak van der Waals interactions. Experiments were performed under ultrahigh pressure and low temperature (5 K) conditions using a homemade cantilevered non-contact atomic force microscopy (NC-AFM) system. As the first step of tip sharpening, the focus is on the controlled extraction of individual clusters. The experimental results show that controlled extraction of individual clusters induces a change in tip sharpness, which affects the long-range force of tip detection. Cluster extraction caused a significant increase in effective tip radius (*R*_T_) and nano-tip height (*z*_0_). They are suggesting that clusters remain attached to the tip after extraction. These findings raise the question of whether the iterations of the extraction process lead to a columnar growth of the clusters, i.e., a more minor contribution of vdW interactions to the total force. To address these issues, Jiménez-Sánchez et al. subjected a blunt tip to multiple clustering extractions and characterized the extracted tip using *F*(z) spectra. As shown in Figure 1a and Figure 1b, the *F*(z) curve and the high-resolution NC-AFM image were used as criteria to test the conditions of the constructed nano-tip. It can be seen from the Figure 1 that the tip remains intact, and the atomic and molar periodicity in the images is resolved with high quality. This work shows that AFM tips sharpened by extracting individual metal nanoclusters can significantly reduce vdW interactions between the tip and the sample and improve spatial resolution. This type of nano-tip is exceptionally robust and can detect different samples. It is expected that tip functionalization methods can achieve enriched species. Sharpening methods can achieve enriched tip functionality.

After preparation, metal nanoclusters have been widely used to detect metal ions, proteins, nucleic acids, and other substances, but few have been done to detect pesticides. Huang et al. [26] used metal nanoclusters to establish a rapid detection method for glyphosate, dimethoate, ethion, methylphosphine and carbaryl. A novel fluorescent "on-off-on" probe was constructed to detect the organophosphorus pesticide glyphosate. Under strongly alkaline conditions (pH is approximately 12), the P–S bonds of ethion, dimethoate, and phorate were broken. The hydrolysis products contain sulfhydryl groups that bind to the silver atoms on the surface of the silver nanoclusters. The formation of non-fluorescent polymers resulted in a significant quenching of the fluorescence of the silver nanoclusters. Cu^2+^ effectively quenched the fluorescence of DNA-templated silver nanoclusters (DNA-AgNCs), while the strong chelation between glyphosate and Cu^2+^ restored the fluorescence of the nanoclusters. The method showed good linearity in the concentration range of 1 ng/mL–50 ng/mL, and the detection limit reached 0.2 ng/mL. In addition, the method was also used for the determination of glyphosate in tap water samples with good recovery results. The assay has the advantages of high sensitivity and good selectivity.

Compared to the above-proposed metal nanoclusters, gold-based bimetallic nanoclusters have the advantages of metal nanoclusters while their fluorescence quantum yields and stability are improved to a larger extent. Gold-silver bimetallic nanoclusters (AuAgNCs) exhibit enhanced luminescence efficiency and photostability due to Förster resonance energy transfer (FRET) and synergistic effects. Zhang et al. [27] synthesized highly fluorescent gold-silver bimetallic nanomaterials with stable fluorescence properties by a simple one-pot method using thiol salts as ligands. TPN-AuAgNCs were synthesised using tiopronin (TPN) as ligand, and the clusters emitted strong red fluorescence with good burst selectivity for Fe^3+^ after Ag^+^ doping. The fluorescence was restored after further adding ascorbic acid (AA) based on a redox reaction. A rapid detection method for Fe^3+^ and AA was established and successfully used to detect Fe^3+^ and AA in human serum. The DMT-AuAgNCs were successfully prepared using 4,6-diaminopyrimidine-2-thiol (DMT) as ligand, and the obtained clusters showed solid red light at 700 nm and sensitive burst selectivity for I^−^. The fluorescence was recovered to a certain extent by further adding Ag^+^. A rapid method for detecting I^−^ and Ag^+^ was established and successfully applied to detect I^−^ and Ag^+^ in actual samples. Gold and silver bimetallic nanoclusters' morphology, properties, and structure were analyzed by various characterization means. The target analytes with specific recognition of the materials were selected, a fluorescence analysis platform for this material was constructed, and the fluorescence response mechanism was further explored to construct a highly selective and sensitive silver-doped gold-based bimetallic nanocluster fluorescent probe.

#### Metal nanowire probe

Nanowires have become a hot research topic as high aspect ratio structures [28–30]. In addition to their plain geometry, the robustness and durability of nanowire scanning probes are also crucial. Due to their unique properties in electrical, magnetic, and optical research fields [31–33], metal nanowires have been prepared by various methods and applied in various fields by domestic and foreign scholars in recent years.

Tay et al. [34] developed a technique based on field emission-induced growth by growing a single metal nanowire at the AFM tip and forming vertically aligned nanowire probes on AFM cantilever beams of different types and force constants. This type of probe has properties such as a high aspect ratio and robustness. This technique based on field emission-induced growth has been maturely applied to produce tungsten nanowire probes. The produced probes have high reproducibility and consistency. Experiments using tungsten nanowire probes have shown that the resulting AFM images are of high resolution because of the tip's high spreading ratio and exceptional sharpness. This technique can be applied to other types of metal nanowire probes, and with further development, composite nanowire probes can be generated by different precursor gases. The tungsten nanowire probes have a diameter of about 5–10 nm and consist of a 3–6 nm low-carbon-containing material wrapped around a tungsten metal core. The technique successfully prepares metal nanowire probes with a tiny radius of curvature (1–2 nm) and high aspect ratios (lengths of 100 nm–1.5 μm), enabling the successful characterization of specific single-walled channel structures.

A faster, one-step technique for growing gold nanowires at the tips of commercial conductive AFM is proposed compared to the above methods. Bakhti et al. [35] grew gold nanowires at the tips of conductive AFM nanoprobes by electroreduction direct deposition based on the deposition of bias-assisted electroreduced gold ions in mesoporous silica films on a conductive substrate. It works in the air with high relative humidity (RH) at room temperature and leads to the growth of fine wires attached to the tip with tens to hundreds of nanometers. Under room temperature conditions with high RH, the gold ions loaded in the specific substrate of the mesoporous silica layer are biased, and the gold ions therein migrate upward and accumulate at the tip site of the probe, forming gold nanowires. The length of the gold nanowires can be effectively tuned in the range of 10–100 nm with a minimum radius of curvature of 3 nm with different applied bias pressures. The resulting gold nanowire probes are chemically inert and have a high lateral resolution. Such gold-functionalized tips can be applied to various spectroscopic and imaging techniques with nanoscale resolution, such as tip-enhanced Raman spectroscopy and fluorescence microscopy. They can open new avenues for characterizing nano-objects and make it possible to study chemical and physical phenomena occurring at the nanoscale.

Following the preparation and application of monometallic nanowire probes, Fang et al. [36] proposed a new probe preparation method based on gold and silver nanowire optical waveguides, which uses the direct coupling of optical fiber and gold and silver optical waveguides to prepare the tip, synthesizes gold or silver nanowires by sol-gel method, etches the hollow optical fiber or pulls the cone by fusion method to make the tip diameter about one μm with a micro-manipulation syringe. The gold and silver nanowires are sucked into the tube at one end, and then the fiber tube is fused with the gold and silver nanowires. When the laser is introduced from one end of the fiber, the light propagates from the fiber to the gold and silver nanowires, which excites the surface plasma propagating on the gold and silver nanowires, and the surface plasma propagates to the tip of the nanowires when it becomes coupled with light. This method of using hollow fiber or glass capillary with gold and silver nanowires is directly coupled to the preparation of the probe, and the traditional method of fiber erosion across the film is more straightforward. The preparation of the probe can be directly used as a scanning near-field optical microscope scanning needle tip. Moreover, the probe prepared by this method can also be used as a nanolight source or nanoscalpel to manipulate and operate on cells directly.

### Carbon nanotube probe

Carbon nanotubes (CNTs) are considered an ideal AFM tip material due to their small diameter, high aspect ratio, mechanical robustness, large Young's modulus, and well-defined structure [37–38]. Due to these unique properties, CNT-AFM probes offer a longer lifetime, high spatial resolution, and unprecedented sensitivity than their conventional silicon counterparts. Carbon nanotubes are generally prepared by assembled and grown methods.

#### Assembled carbon nanotube probe

Nishijima et al. [39] proposed a multistep assembly method to fabricate carbon nanotube tips by developing a well-controlled procedure to fabricate scanning probe carbon nanotube tip microscopes. The process consists of three steps: purification and alignment of nanotubes using electrophoresis; transfer of target nanotubes to Si tips under scanning electron microscopy; and attaching nanotubes to Si tips by carbon deposition. The strong adhesion of carbon deposition produces nanotube tips capable of surviving multiple surface collisions. The ability to image the fine structure of double-stranded DNA molecules demonstrates the resolution of these nanotube tips and the potential to elucidate the structural features of biological specimens. The method involves the growth, purification, and movement of carbon nanotubes into a cartridge and, finally, the transfer of the carbon nanotubes to the tip under an electric field. The nanotube tips produced by this method have strong adhesion and mechanical stability.

Since the above methods require scanning electron microscopy (SEM) monitoring throughout the transfer process, the process is relatively time-consuming. Hafner et al. [40] proposed a new method to extract vertically aligned carbon nanotubes based on the surface layer of the substrate. This method extracts single-walled carbon nanotubes (SWNTs) on the substrate surface based on the observation of micromachined tips by first fabricating vertically aligned SWNT substrates with isolation effects, followed by imaging with commercially available silicon tips. Individual SWNTs are extracted from the substrates to produce superior SWNT probes in this process. This type of tip can be applied for etching by adjusting the length of the nanotubes under 2 nm control. In fluid and air, this type of individual SWNT tip can be imaged stably with high-resolution images. Therefore, this tip type can have deeper applications in the nano field. However, due to large van der Waals forces, multiple carbon nanotubes tend to attach to AFM probes.

Based on the above approach, a simple method was proposed to stabilize carbon nanotube fibers. Slattery et al. [41] prepared carbon nanotube probes by solvent evaporation or dielectrophoresis, the first time a solvent evaporation method was used. From the 13 probes produced using these methods, the CNT-modified nanomaterials showed very high aspect ratios and good alignment. Due to the poor stability of the carbon nanotube fibers, many probes were initially not possible for AFM imaging with the prepared carbon nanotube probes modified. However, Slattery et al. developed a method to straighten, shorten, and strengthen carbon nanotube fibers for thorough AFM imaging and set low amplitude points. Slattery et al. prepared cost-effective carbon nanotube-enhanced probes capable of imaging at high resolution using a stable procedure. However, variations in carbon fiber length and straightness affected the scanning performance.

#### Grown carbon nanotube probe

Compared to assembly methods, direct growth of carbon nanotubes by chemical vapor deposition (CVD) allows for increased bond strength between CNT tips and AFM probes. A pore growth method was used by Hafner et al. [42]. The method uses AFM imaging in contact mode to flatten the silicon tip, followed by hydrogen fluoride oxidation at the anode to form nanopores of 50–100 nm in diameter. After electrodeposition, the carbon nanotubes are grown by CVD at 750 °C. If the carbon nanotubes do not grow in a suitable trend, they are removed by oxidation. The CNT tips are then grown again by CVD. The CVD nanotube tips can be generated again when the reaction reaches ten minutes. Usually, this type of nanotube is very long and cannot be used as a tip. Therefore, it is shortened by the AFM technique and the tip is used for imaging to clearly observe a tube with a length of 480 nm protruding from the tip of Si. The average diameter of the tip generated under this method is 10 ± 5 nm. Transmission electron microscopy confirmed that this tip type is a multi-walled nanotube (MWNT) formed by ordered graphene walls. In any case, however, the pore growth method does not allow the growth of a single carbon nanotube at the optimal location on a flat tip. Due to the considerable length, a subsequent cutting process is required.

Since the perpendicularity of the carbon nanotube probe is difficult to achieve in the above mentioned pore growth method, Edgeworth et al. [43] demonstrated a catalytic CVD (cCVD) growth method that generates a high-density network of CNTs on the sidewalls of the cone tip to help anchor the carbon nanotubes protruding from the tip. With the aim of simplicity in design and method of operation, the growth method does not use plasma or electric field as an enhancing factor to obtain effective tips, nor does it generate catalyst patterns in advance. In order to proceed safely and cost-effectively, the method uses mainly ethanol as the carbon source with a 4% flow gravity of hydrogen gas. Notably, using this tip array allows the growth method to be further optimized to produce the highest percentage of prominent carbon nanotube tips. This type of process strategy is used to produce CNT tips in wafer-scale AFM. By identifying and manipulating the key growth conditions that control the density and length of carbon nanotube growth; i.e., the amount of Co catalyst and CNT growth time, it is possible to switch between ring protruding carbon nanotubes and carbon nanotubes protruding directly from the tip with a high degree of selectivity. However, this growth method does not allow accurate catalyst placement and often produces ring-like tips.

Based on the above method, Cheng et al. [11] proposed a simple method to generate CNT probes with a high aspect ratio. The method focuses on adjusting the trigger threshold and thus the amount of growth solution attached to the tip accordingly to fabricate individual CNTs. The success rate is well over 93%, and the generated CNT probes do not require further cutting and are of suitable length. This controllable method has great adhesion for CNT probes. The growth of individual CNTs at the tip was achieved when the trigger threshold was controlled in the range of 0.25–0.50 V. The properties and functionality of such tips were verified using samples with high aspect ratios. The imaging results showed higher spatial resolution and prominent tips with CNTs with aspect ratios greater than 10 compared to standard AFM probes. Using such CNT probes for scanning complex nanostructures could solve the long-standing problem of mapping complex nanostructures.

#### Composite probe

Manufacturing nanoprobes that meet the requirements for cutting-edge mechanical properties, dimensions, and morphology is practically challenging. Modern technology has made it possible to produce macroscopic materials with certain functions by combining several components into composite materials. This direction has also developed concerning nanomaterials. Carbon nanotubes are compounded with related materials to produce probes, and the analytical performance of this type of probe is better than that of carbon nanotube probes alone.

Nakabayashi et al. [44] proposed using amorphous carbon matrix (shell) covered nanotubes to produce reinforced carbon–carbon composite nanotools; combining amorphous carbon with the extreme mechanical properties of CNTs can facilitate the production of nanotools with high aspect ratios. Inappropriate properties such as vibration and flexibility can be controlled without compromising the carbon nanotubes' aspect ratio, strength and size. The mechanical response of these composite beams under bending is studied in molecular dynamics simulations and nanomanipulation experiments. The behavior of this system has been studied at both theoretical and experimental levels. AFM probes based on these C–C composites' high aspect ratio tips produced highly high image resolution and good wear resistance, with no degradation of tip or image quality observed after 400 images had been acquired.

Compared with the above composite probes, the combination with metal nanowires also performs well. Clark et al. [45] presented a novel scanning probe for mechanical and electronic characterization of probe microscopy. A newly developed controlled area plating method was used. The method uses microwave plasma to enhance the growth of carbon nanotubes in chemical vapor deposition. This process resulted in the growth of coaxial palladium nanowires/carbon nanotube composite structures (PdNWCNTs) using catalyzed palladium films deposited only near the tip of AFM suspensions in commercial tapping mode. The experimental results show that the PdNWCNT probe is sufficient for standard percussive mode AFM imaging, and the growth of PdNWCNT does not significantly decrease the cantilever spring constant and cantilever mass factor. PdNWCNTs showed better performance than standard CNTs in some SPM applications. For example, because it is difficult to form good ohmic contact CNTs, the existence of the internal Pd nanowires will help probe more surface conductivity measurement, the nanowires with nanostructure or form good ohmic contact to the characterization of surface, external carbon nanotubes provide higher mechanical stability, can make the probe after many times of measuring still keep its original shape. Low-resistance ohmic contacts between the metal surface and the PdNWCNT probe were confirmed. Moreover, repeated current flow and surface contact did not cause any damage to PdNWCNTs, indicating that the PdNWCNT probe is suitable for multi-probe conductivity measurement. In the future, such probes will enable previously unexplored conductivity measurements, such as measurements of semiconductor nanostructures or electrical conductivity on insulating substrates.

Conductive atomic force microscopy (C-AFM) can be used to characterize the electrical properties of semi-conductive and conductive materials at the nanoscale. Slattery et al. [46] describe the use of Pt/Ir conductive tips modified with single-walled carbon nanotubes (SWCNTs), a type of tip suitable for use in conductive imaging mode with high-sensitivity current acquisition AFM, which can also be applied to worn conductive coatings and regenerated broken tips. The Pt/Ir cantilever was modified with small bundles of SWCNTs by a manual attachment process and fixed using a conductive Pt pad. AFM images of the current and topography of the nanomaterial samples and non-homogeneous polymers were collected using this type of tip. The attachment process produces a tip with stability and higher sensitivity during image acquisition. The smaller tip diameter produces a higher peak force, resulting in very sharp images being collected. In general, if good images are to be achieved, it is at the expense of reduced feedback stability. This is partly due to the increased wear of the tip and electrostatic effects. SWCNT-modified tips offer feedback stability and higher current sensitivity, and SWCNT has good wear resistance. The overall performance of SWCNT probes shows that they can be produced at a lower price and meet all the criteria required for a high-quality AFM conductive probe.

### Colloidal probe

Colloidal probes (CPs) [47] can simulate the determination of forces between many types of colloidal particles, such as silica-alumina cellulose, and have several advantages for accurate force measurements: tunable and well-characterized radii; higher averaging power (at the cost of spatial resolution) and sensitivity to weak interactions; well-defined interaction geometry (spheres in the plane), allowing for analytical models for accurate and reliable data fitting. Colloidal probes provide an excellent detection tool for understanding interactions in various domains to study adhesion phenomena, particle-surface interactions, mechanical properties, suspensions, liquid dynamics, and boundary slips.

#### Colloidal gold probe

Colloidal gold particles [48–49] have the advantage of stable adsorption of proteins without significant changes in the biological activity of proteins, so they are widely used as an immunolabeling probe in immunocytochemistry, and with the rapid development of molecular biology, colloidal gold labeling techniques are used as a means to perform precise localization of biological macromolecules such as cell surface and intracellular polysaccharides, proteins, peptides, antigens, hormones, and nucleic acids. They are gradually developing into an essential means to study gene function.

AFM direct force measurements are mainly due to the colloidal probe technique's defined interaction geometry and versatility. Karg et al. [50] proposed a method that could develop the colloidal probe technique in the direction of electrochemistry. This preparation method allows the selection of many colloidal particles containing electrochemical activity as probes. The colloidal gold particles in the experiments based on this method were produced by rapid short-circuiting of two gold wires on a microscope slide in a sealable glass container. At the same time, the current limiter was set to a maximum value, and an external voltage of 30 V was applied to collect the particles produced in the sparks on the slide, resulting in a broad particle size distribution, which was observed to be in the range of 2–80 μm. The polydispersity due to evaporation in the spark helps to select particles of suitable diameter size. The evaporation of sparks is a universal process that can be applied to basically all metals suitable as electrodes, such as copper, silver, platinum, etc. Thus, it is also possible to prepare electrochemical colloidal probes (eCPs) with other metals. eCPs under electrochemical control of colloidal probes have potential in various research areas such as adhesion science, tribology or long-range interactions. eCPs combine the versatility of electrochemistry and CP technology. They are more accessible in terms of samples, either as a second electrode under open circuit conditions, under double constant potential control or as an insulating surface. In the future, there is excellent scope for eCPs in applications such as electrochemical impedance spectroscopy, where the AC potential is applied to eCPs, and where such first tests are promising. Most importantly, eCPs are extremely stable and can be applied to electrochemically controlled tribology or nanoindentation experiments. With these foundations, eCPs will likely be critical in bioelectronic applications or battery research.

Zhang et al. [51] developed qualified colloidal gold probes labeled with monoclonal antibodies to hepatitis B surface antigen (HBsAg Mab), and l5 nm colloidal gold was prepared by using the trisodium citrate reduction method; the prepared colloidal gold was identified by transmission electron microscopy and UV spectrophotometer for size and uniformity. The amount of colloidal gold-labeled HBsAg Mab protein was determined by the CVAI curve; the probe was identified by spot immunosorbent assay. The prepared 15 nm colloidal gold particles were homogeneous; the maximum absorption wavelength was 518 nm with narrow peak width in the UV spectrophotometer 400–700 nm scan; the purified HBsAg Mab concentration was 65 mg/mL; the optimal protein protection amount was 32.5 μg per mL of colloidal gold at pH 8.2; the quality of the probe was qualified by spot immunosorbent assay. The probes were qualified by speckle immunosorbent assay and could be stored for three months. The quality of the prepared HBsAg Mab colloidal gold probes was satisfactory and provided a means for further studies.

Based on the above trisodium citrate reduction method, Liu et al. [52] prepared single-chain antibodies against *Listeria monocytogenes* (Lm) with an *E. coli* expression system, prepared colloidal gold by the trisodium citrate method, used colloidal gold as a tracer, optimized the preparation of colloidal gold probes, combined with purified scFv to prepare colloidal gold probes, and subjected to an immunoassay. The activity of the colloidal gold probe was determined by immunofiltration. The results showed that the colloidal gold probe was highly specific when applied to the immunodiafiltration method, and positive samples could be determined by direct color development of the colloidal gold probe. The preparation of a high-quality colloidal gold probe was a prerequisite for the detection of Lm, in which the pH value and the amount of labeled scFv were two critical influencing factors. Therefore, the study was optimized for both factors. Also, the colloidal gold probe preparation had to be followed by activity characterization to ensure the usability of the colloidal gold probe. The results showed that the detection limit of Lm was about 10^7^ CFU/mL when the prepared colloidal gold probe was used in the immunodiafiltration method. It only took about 10 minutes to complete the detection process. The method is simple, rapid, and accurate and can be used to detect Lm in food. However, the probe lost its activity after storing at 4 °C for 28 d. This may be due to the small molecular mass, simple structure, and poor stability of the scFv relative to the intact antibody molecule. Therefore, subsequent research is needed to improve the stability and enhance the ease of use of the colloidal gold probe.

To improve the stability and sensitivity of this type of probe, Miao et al. [53] proposed a homogeneous and "non-"fluorescent aptamer detection method for chloramphenicol based on a vesicular quantum dot-gold colloid composite probe. They successfully developed a fluorescence resonance energy transfer (FRET) based on chloramphenicol (CAP) detection in food. Vesicular nano tracers were prepared by labeling single-stranded DNA-binding proteins on single-stranded -cdse/ZnS quantum dots. This type of tracer has a significant signal amplification effect. Vesicle composite probes were generated using aptamer-labeled SSB/L-QD and nanogold (Au-Apt). Since Au-aptas receptors burst the signal of SSB/L-QD, this type of probe cannot fluoresce "off". To solve this problem, the CAP is added to the complex probe solution, and the aptamer is released after binding to the CAP, which can change the "off" state to the "on" state in the tracer. The experimental results show a good linear response to CAP with a detection limit of 0.3 pM starting from 0.001 nM. 88 nm size vesicle probes have a strong signal amplification ability. More quantum dots can be labeled within the double phosphorus lipid membrane. The detection process is straightforward and does not require pretreatment. The fluorescence signal can be easily switched from "off" to "on" after the target is added to the reaction system. Therefore, the method is suitable for the field detection of antibiotics in liquid foods. More importantly, the proposed homogeneity method has been successfully applied to actual milk samples. This novel homogeneity method with high sensitivity and selectivity is a powerful tool for biomedical research and food detection.

#### Bifunctional probe

Yang et al. [54] prepared a novel bifunctionalized colloidal gold nanoprobe and investigated its specificity due to the excellent performance of bifunctional probes for food pathogen detection and nucleic acid analysis. A bifunctionalized colloidal gold nanoprobe (burgundy color of the recognition probe) was made by assembling selected disulfide molecules and a 23mer oligonucleotide fragment specific for the HA gene of influenza A virus (H1N1 subtype) onto the surface of colloidal gold particles; a capture probe was made by coupling another specific H1N1 oligonucleotide fragment using magnetic microspheres as solid-phase support; both were bound to the target DNA (exact match DNA) to form a colorless nucleic acid probe. The two are combined with the target DNA (exact match DNA) to form a colorless capture probe-target DNA-recognition probe ("sandwich" complex) and measured by matrix-assisted laser resolved ionization time-of-flight mass spectrometry (MALDI–TOF MS). Ultra-pure water (as a blank control), perfectly matched DNA, incompletely matched DNA, and two DNA fragments with only one base mismatch were added to the system as targets to observe the system's color change and investigate the specificity of the probes by mass spectrometry. The results showed that the capture and recognition probes could only bind to the perfectly matched DNA to form a sandwich complex, and the color of the reaction system changed from burgundy to colorless. The reaction system did not change in color (it still appeared burgundy) after sufficient washing and heating, and no signal was detected by mass spectrometry (*m*/*z* 693). This indicates that the specificity of the colloidal gold nanoprobe is high enough to distinguish a target with only one base mismatch. This study developed a novel self-assembled bifunctionalized colloidal gold nanoprobe with a simple preparation process with high sensitivity and high specificity. This colloidal gold nanoprobe will have a wide range of applications in nucleic acid analysis, especially in oligonucleotide polymorphism typing for early disease diagnosis.

To achieve quantitative detection of pathogenic microorganisms and illegal additives in food, Huang et al. [55] established a method for quantitative detection of CAP by immunochromatographic techniques based on background fluorescent burst probes by preparing colloidal gold particles with a particle size of about 30 nm and combining them with monoclonal antibodies to CAP to prepare burst probes. When there is a test substance in the sample, the test substance competes with the antigen wrapped on the chromatography paper to produce a red detection line (T line) and a quality control line (C line) and causes the background fluorescence at that position to be burst, which is read by the instrument. The instrument read the background fluorescence value (T0) and the T-line fluorescence value (T1), and T0/T1 was calculated for the quantitative analysis of the analytes to be measured. The feasibility of the method was verified by the spiked recovery method. In this study, a novel quantitative immunochromatographic assay for small molecules was successfully designed with a linear range of 0.1–2.0 ng/mL for CAP, a 100% detection rate, and 95.8%–109.6% recovery for spiked samples, which is rapid, simple, economical and accurate and can be easily applied.

Colloidal probes in the types mentioned above are usually non-conductive spheres. Therefore, conductive gold spheres were introduced. Daboss et al. [14] proposed a novel conductive colloidal BDD-AFM probe, which is particularly suitable for complex SPM experiments based on BDD properties. The probe is mainly a conductive BDD sphere attached to an embedded microelectrode at the end of a tipless cantilever beam. The AFM cantilever beam is completely insulated except for the contact pad on the chip, the BDD. The developed spherical BDD-AFM-SECM probes can be applied to complementary SPM experiments. The robustness of this class of bifunctional probes and the excellent electrochemical properties make it a versatile tool that can be applied to various scanning probe studies, such as tip integration for chemical or biological sensing.

#### Large size colloidal probe

Large colloidal probes are widely used to study the mechanics of samples with weaker interaction forces and local variations over a large contact range, and large CPs enable a better comparison of the results achieved in AFM compared to other probes, which can link macromechanics, micromechanics and nanomechanics well under certain conditions.

Conventional AFM probes used for interaction force testing have sharp tips and have a small contact area with the surface contact. To study interaction properties on the surface of micro- and nanostructures, it is necessary to use more significant micron level colloidal probes to characterize the effect of structural features of the micro- and nanostructures on dopa adhesion. Zhang et al. [56] proposed using the colloidal probe technique to study the interaction between dopa and the surface of nano-, micron, and micro- and nanocomposite structures. In this study, ten μm diameter silica spheres were selected as the particles for the colloidal probe and dispersed into an aqueous solution and sonicated for five minutes. A small amount of the dispersed liquid was removed and added dropwise to a clean mica flake surface and dried naturally. This experiment used a DNP-10 AFM probe without a tip (Bruker, USA) to prepare the colloidal probe. Then a small amount of epoxy resin adhesive mixed with a 1:1 ratio was glued to the tip of the AFM probe under the high optical field of the AFM. The individual silica spheres on the mica flakes were glued immediately. A silica colloidal probe was prepared. The adhesion properties of each nanostructured surface were tested using end group-modified dopa AFM colloidal probes. It was found that the nanostructured surface of evaporated and plated 200 nm thickness eicosanoid crystals had low adhesion. The adhesion force of dopa to the nanostructured surfaces of 1, 3, and 5 μm height was lower than that of the nanostructured surfaces of 200 nm thickness of eicosanoid crystals, indicating that the nanostructured surfaces have strong resistance to dopa adhesion. This composite surface also has strong hydrophobicity and excellent resistance to water adhesion compared with the arid gold lily's silicon sharkskin-like micron structure surface.

The colloidal particles in the above method present a relatively stable colloidal suspension in water or ethanol solvent. The colloidal particles in the suspension can self-assemble into two- and three-dimensional colloidal crystals of various shapes under certain conditions. In the process of self-assembly, such fibrous colloidal crystals are small in size and usually micrometer in length due to the weak interaction forces between colloidal particles, and the introduction of templates requires precise operating conditions, leading to higher preparation costs, so it is necessary to find a template-free method to prepare regular large-size colloidal fibers. Xie et al. [57] reported a convenient and straightforward large-size colloidal fiber preparation, proposing a method to self-assemble colloidal particles into regular fibrous colloidal crystals by controlling the crack growth. It was shown that the crack expansion direction is always perpendicular to the "curing front" (i.e., the dividing line between the curing part and the solution part), therefore, the crack expansion direction can be effectively controlled by controlling the direction of the "curing front", which is the theoretical basis for the preparation of colloidal fibers. This is the theoretical basis for the preparation of colloidal fibers. Many factors influence particle self-assembly and crack extension. Therefore, the controllability of colloidal fiber morphology can be achieved by changing the experimental conditions. When the colloidal particles are self-assembled into colloidal films, regular parallel-like crack patterns are generated in the colloidal films by controlling them, thus causing the colloidal films to split to form regular fibrous colloidal crystals. The morphology of the colloidal fibers can be effectively controlled by controlling the dispersion components, temperature, particle concentration, and other factors, including the length, width, and thickness of the colloidal fibers. In addition, high-temperature calcination can increase the interaction force between particles, thus enhancing the mechanical properties of colloidal fibers. Due to the rough surface structure of colloidal fibers, we made them into surface Raman enhanced scattering (SERS) probes and used them to detect bisphenol A (BPA) in water. The experimental results showed that the colloidal fiber probes could detect 10^−8^ M BPA in water. This method of preparing colloidal fibers is simple and easy to operate and can be prepared on a large scale, providing new opportunities for many fields. In practical applications, due to the surface structure of colloidal fibers, these fibers can be used as SERS sensor detection for trace detection of BPA.

Although colloidal probes have many excellent properties, there are some problems in calibrating such probes, especially large colloidal probes, where the intrinsic static deflection sensitivity and spring coefficient need to be verified. Chighizola et al. [58] proposed an accurate method for calibrating large CPs. This method is applied to calibrate the intrinsic spring constant *k*_TL_ of a topless cantilever beam by the thermal noise method when prepared in the chamber, after which FC is obtained on the substrate to measure the deflection sensitivity, and the position of the loading point is measured by optical microscopy when the sphere is attached. The process of calibrating the *k*_TL_ into the manifold 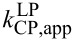 is shown in Equation 1.


[1] $$\begin{array}{c} k_{\text{CP,app}}^{\text{LP}}={\left\{\left[1-\frac{3R/L}{2(1-\Delta L/L)}\tan\theta \right]\cos^{2}(\theta )\right\}}^{-\text{1}}{\left(\frac{L}{L-\Delta L}\right)}^{3}k_{\text{TL}}\\ ={\left\{\left[1-\frac{3R/L}{2(1-\Delta L/L)}\tan\theta \right]\cos^{2}(\theta )\right\}}^{-1}k_{\text{CP}}^{\text{LP}} \end{array}$$

In this method, Chighizola et al. verified that adding a large colloid at the end of a topless cantilever beam increased the quality factor and significantly reduced the resonant frequency. This type of probe can better simulate a single-mode spring-mass system. Although some adjustments are made to its cantilever dynamics, the calibration accuracy of CPs does not fluctuate when the additional mass of the ball is the same as the cantilever. In the type of large CPs, applying the homogeneity theorem for topless cantilever beams to Equation 1 can achieve accurate calibration. These studies will give further impetus to the application of CPs in spectral dynamics.

## Conclusion and Outlook

Probe technology has been applied in physics, chemistry, biology, and nanotechnology in the past few years, and outstanding results have been achieved. Metal, carbon nanotube, and colloidal probes have been widely used. Metal plating probes are generally suitable for high-resolution or high conductivity test experiments. Carbon nanotube probes have a large aspect ratio and good wear resistance, thus can better avoid artefacts and more accurately reflect the actual shape of the vertical position of the sample, and are suitable for high-resolution test experiments. Colloidal probes are mainly used to study colloidal interactions. They can simulate the determination of the forces between various types of colloidal particles such as silica-alumina cellulose, providing a good detection tool for a better understanding of interactions in various fields. This paper reviews the latest research results on metal, carbon nanotube, and colloidal probes. The differences in spatial resolution, sensitivity, imaging, and other performance aspects are compared. The advantages and disadvantages of the improved probes compared with standard probes are analyzed in Table 1.

**Table 1:** Comparison of probe tip materials.

AFM probe tip material		Advantages	Disadvantages

metal probe	metal nanocluster probe	excellent photostability, large Stokes shift, low toxicity, and improved spatial resolution	susceptible to environmental interference and unable to observe color changes with naked eyes
	metal nanowire probe	high aspect ratio structure, robustness, and durability	there are some problems in the preparation process, such as poor reproducibility, low yield, uneven morphology, and long reaction time
carbon nanotube probe	assembled carbon nanotube probe	suitable for small-batch, high-quality manufacturing	low efficiency and time-consuming
	grown carbon nanotube probe	high bonding strength, high selectivity, suitable for mass production	easy to produce a circular tip
	composite probe	good analytical performance	the preparation process needs to be optimized
colloid probe	colloidal gold probe	the adsorbed protein was stable, but the protein's biological activity was not significantly changed	usability needs to be improved
	bifunctional probe	high sensitivity and specificity, easy to popularize and apply	application to be promoted
	large size colloidal probe	suitable for studying fragile interactions	probe calibration needs to be improved

The development of AFM technology strongly depends on micro-cantilever probe preparation technology. Hence, developing probes with stable performance, high resolution, long life, and broader applications is a hot spot and complex area of current research in AFM technology. Therefore, in the field of AFM probes in the future, ultra-fine ultra-tip and ultra-long-life probes may be developed to improve the resolution and lifetime of electrical and magnetic energy probes. The nanosizing of probes, especially the modification of carbon nanotubes and functional nanomaterials, will significantly improve the performance of probes and further promote the broader and deeper application of SPM. It will significantly impact society in life science, drug discovery, and medical diagnosis.
